# Obesity Attenuates Ventilator-Induced Lung Injury by Modulating the STAT3–SOCS3 Pathway

**DOI:** 10.3389/fimmu.2021.720844

**Published:** 2021-08-20

**Authors:** Shih-Wei Wu, Chung-Kan Peng, Shu-Yu Wu, Yu Wang, Sung-Sen Yang, Shih-En Tang, Kun-Lun Huang

**Affiliations:** ^1^Division of Pulmonary and Critical Care, Department of Internal Medicine, Tri-Service General Hospital, Taipei, Taiwan; ^2^Graduate Institute of Medical Sciences, National Defense Medical Center, Taipei, Taiwan; ^3^Institute of Aerospace and Undersea Medicine, National Defense Medical Center, Taipei, Taiwan; ^4^Division of Nephrology, Tri-Service General Hospital, National Defense Medical Center, Taipei, Taiwan

**Keywords:** obesity, ventilator-induced lung injury (VILI), suppressor of cytokine signaling 3 (SOCS3), hesperetin, WNK lysine deficient protein kinase 4 (WNK4), alveolar fluid clearance (AFC)

## Abstract

**Background:**

Ventilator-induced lung injury (VILI) is characterized by vascular barrier dysfunction and suppression of alveolar fluid clearance (AFC). Obesity itself leads to chronic inflammation, which may initiate an injurious cascade to the lungs and simultaneously induce a protective feedback. In this study, we investigated the protective mechanism of obesity on VILI in a mouse model.

**Methods:**

The VILI model was set up *via* 6-h mechanical ventilation with a high tidal volume. Parameters including lung injury score, STAT3/NFκB pathway, and AFC were assessed. Mice with diet-induced obesity were obtained by allowing free access to a high-fat diet since the age of 3 weeks. After a 9-week diet intervention, these mice were sacrificed at the age of 12 weeks. The manipulation of SOCS3 protein was achieved by siRNA knockdown and pharmaceutical stimulation using hesperetin. WNK4 knockin and knockout obese mice were used to clarify the pathway of AFC modulation.

**Results:**

Obesity itself attenuated VILI. Knockdown of SOCS3 in obese mice offset the protection against VILI afforded by obesity. Hesperetin stimulated SOCS3 upregulation in nonobese mice and provided protection against VILI. In obese mice, the WNK4 axis was upregulated at the baseline, but was significantly attenuated after VILI compared with nonobese mice. At the baseline, the manipulation of SOCS3 by siRNA and hesperetin also led to the corresponding alteration of WNK4, albeit to a lesser extent. After VILI, WNK4 expression correlated with STAT3/NFκB activation, regardless of SOCS3 status. Obese mice carrying WNK4 knockout had VILI with a severity similar to that of wild-type obese mice. The severity of VILI in WNK4-knockin obese mice was counteracted by obesity, similar to that of wild-type nonobese mice only.

**Conclusions:**

Obesity protects lungs from VILI by upregulating SOCS3, thus suppressing the STAT3/NFκB inflammatory pathway and enhancing WNK4-related AFC. However, WNK4 activation is mainly from direct NFκB downstreaming, and less from SOCS3 upregulation. Moreover, JAK2–STAT3/NFκB signaling predominates the pathogenesis of VILI. Nevertheless, the interaction between SOCS3 and WNK4 in modulating VILI in obesity warrants further investigation.

## Introduction

Acute lung injury (ALI) and its serious form, acute respiratory distress syndrome (ARDS), remain associated with high mortality and morbidity in current critical care. Despite many efforts, the therapeutic options for ARDS remain limited and mostly supportive (1). Among them, the development of a lung-protection strategy in mechanical ventilation forms the basis of the fight against ARDS (2–5). However, mechanical ventilation itself carries the risk of causing ventilator-induced lung injury (VILI), either significantly or insidiously (6–8).

Obesity is a growing public health issue that impacts populations worldwide because of its high prevalence, comorbidity, and medical costs. Numerous comorbidities and complications of obesity have been identified, such as diabetes, cardiovascular disease, cancer, and osteoarthritis (9–11). Similarly, obesity increases the risk of developing ARDS and the length of hospital stays (12). However, surprisingly, a similar or even lower mortality risk for ARDS was observed in patients with obesity compared with individuals without this condition in several clinical studies (13–16). Other than these observations, clinical prospective studies focusing directly on the relationship between obesity and ALI/ARDS are still lacking, probably because of the presence of multiple confounding factors in the clinical setting and ethical issues. Nevertheless, a growing number of basic studies, including those using cell and animal models, are focusing on identifying possible mechanisms and causal relationships between obesity and ALI, mostly VILI (17–21). Considering the beneficial effect of low tidal volume ventilation on ARDS (2), it is reasonable to hypothesize that obesity decreases the mortality of ARDS by attenuating VILI.

The suppressor of cytokine signaling 3 (SOCS3) is an overexpressed protein that leads to leptin resistance in patients with obesity (22, 23). Increased baseline expression of SOCS3 in subjects with obesity is supposed to instantly hinder the signal transduction of the signal transducer and activator of transcription 3 (STAT3) and nuclear factor kappa-light-chain-enhancer of activated B cells (NFκB) after a VILI insult, as well as to subsequently alleviate inflammatory cascades. In addition to the inflammatory process, the removal of alveolar fluid after a mechanical insult is another important component of ALI (24, 25). Recent research has revealed an association between WNK lysine-deficient protein kinase 4 (WNK4) and its downstream pathway, including Ste20-related proline–alanine-rich kinase (SPAK) and sodium potassium chloride cotransporter-1 (NKCC1), and alveolar fluid clearance (AFC) and ALI (26–29). Overexpression of WNK4 resulted in the suppression of AFC and the increase of lung edema. However, it remains unknown whether obesity attenuates VILI by modulating the WNK–SPAK pathway.

Therefore, this study was designed to test our hypothesis that obesity and SOCS3 contribute to the attenuation of inflammation and the enhancement of AFC in an obese VILI mouse model.

## Materials and Methods

### Animals and Diet-Induced Obesity

The animals used in this study were cared for according to the National Institutes of Health guidelines (National Academy Press, 1996). Protocols were approved by the National Science Council and the Institutional Animal Care and Use Committee at the National Defense Medical Center (Taipei, Taiwan). Three-week-old male C57BL/6 mice were purchased from BioLASCO Taiwan Corporation (Taipei, Taiwan). The knockout (WNK4^−/−^) and knockin (WNK4^D561A/+^) mice used here were established by Yang et al (30, 31). A previous study reported that WNK4^−/−^ mice exhibited lower NKCC1 expression, whereas the WNK4^D561A/+^ animals showed higher NKCC1 expression. To achieve the obese condition, animals had free access to a high-fat diet (5.1 kcal/g, 61.6% from fat) (**obese mice**) or a normal control diet (4.07 kcal/g, 13.5% from fat) (**nonobese mice**) from the age of 3 weeks. After a 9-week diet intervention, these mice were weighted and grouped for experiments at the age of 12 weeks.

### Experimental Protocols for the VILI Model

Mice from both groups were fixed on a tilting plate with the head up to 60°C, to minimize the mechanical effect of obesity itself. Based on the results of the ARDSNet trial (2), which revealed that a low tidal volume yielded a better outcome, VILI seems to play an important role in the development of ARDS. Here, we used a one-hit model lasting for 6 h, using ventilation as the only hit. Experimental mice were anesthetized (sodium pentobarbital, 70 mg/kg) and mechanically ventilated *via* intratracheal intubation using a volume-controlled ventilator and 100% oxygen. Intratracheal intubation was accomplished by the insertion and fixation of a PE-60 tube over the incised trachea. The nonobese group was similarly anesthetized and intubated, but was allowed spontaneous breathing. High tidal volume ventilation (**HV**, 24 mL/kg; peak pressure, 40 cm H_2_O; frequency, 100/min) for 6 h was performed and compared with animals with spontaneous breathing (control volume ventilation, **CV**). The peak inspiratory pressure (PIP) was calculated by averaging the PIP values recorded over each 10-min period (0–10 min, 175–185 min, and 350–360 min after mechanical ventilation) (**Supplementary Figure 1**).

### Measurement of AFC

AFC was determined using an in-situ mouse-lung model (32–34). At the end of the mechanical ventilation, mouse lungs were inflated with 100% oxygen at a continuous positive airway pressure of 7 cm H_2_O. Subsequently, an instillate containing fluorescein isothiocyanate (FITC)-labeled albumin (Sigma-Aldrich, St. Louis, MO, USA) was perfused into the lungs over 1 min at 12.5 mL/kg of body weight. An alveolar fluid sample (0.1 mL) was collected respectively at 1 and 15 min after instillation. The samples were centrifuged at 3000 g for 10 min, and the fluorescence activity was measured in the supernatant in duplicate. AFC was calculated based on the increase in albumin concentration in the alveolar fluid using the following equation: AFC = (Cf − Ci)/Cf × 100, where Ci and Cf represent the initial and final concentrations of FITC-labeled albumin in the sample at 1 and 15 min, respectively, as assessed by the fluorescence activity measurements.

### Knockdown of SOCS3 *via* the Intratracheal Delivery of a Short Interfering RNA (siRNA)

For the *in vivo* study, a chemically synthesized siRNA against SOCS3 (5′–GGACCAAGAACCUACGCA–3′, Dharmacon, Lafayette, CO, USA) was administered *via* direct intratracheal spraying. This intratracheal delivery was performed 48 h before the experiment started. The knockdown effect of the SOCS3 siRNA was validated *in vitro* in a murine pulmonary epithelial cell line (**Supplementary Figure 2**), and *in vivo* attenuation of SOCS3 expression (**Supplementary Figure 3**).

### Pharmaceutical Upregulation of SOCS3 Expression Using Hesperetin

For practical purposes, overexpression of SOCS3 was achieved using a pharmaceutical method. A previous *in vitro* study has suggested that flavonoids induce the expression of SOCS3 in cells (35). Hesperetin, a common and accessible flavonoid, was chosen to enhance SOCS3 expression *in vivo*. Based on previous publication (36) and preliminary study (**Supplementary Figure 4**), hesperetin at the dose of 40 mg/kg of body weight was chosen and fed enterally 12 h before the onset of mechanical ventilation.

### Sample Collection and Measurements

Mice were sacrificed by cervical dislocation after 6 h of mechanical ventilation or spontaneous breathing. The sternum of mice was incised and fixed for subsequent sample collection. The lungs were removed from the chest cage and weighed to calculate the lung-to-body weight ratio. Total lung weight was also measured as an indicator of pulmonary congestion. Bronchoalveolar lavage fluid (BALF) was collected at the end of the VILI protocol by tying the right main bronchus and irrigating the left lung with two separate 0.5-mL aliquots of phosphate-buffered saline, of which approximately 0.8 mL was recovered consistently. One aliquot of BALF was processed immediately to measure total white cell counts. The other aliquot of BALF was centrifuged at 800 × *g* for 10 min, followed by the measurement of the concentration of proteins in the pellet and cytokines in the supernatant.

The right lung was excised and prepared for the measurement of proteins and cytokines in the tissue. Cytoplasmic, nuclear, and mitochondrial proteins were extracted from right lung tissue using the Nuclear/Cytosol Extraction Kit and Mitochondria/Cytosol Fractionation Kit (Sigma, St. Louis, MO, USA), according to the manufacturers’ instructions. Protein concentrations were measured using a BCA protein assay kit (Sigma, USA), and cytokine concentrations were measured by immunoassay (R&D Systems, Minneapolis, MN, USA). Western blotting was performed according to the instructions of the manufacturer (Cell Signaling Technology, Danvers, MA, USA).

### Lung Histopathology and Lung Injury Scoring

Histology was performed as described previously (34). The lungs cut down were instilled and fixed in 10% neutral-buffered formalin for 24h. The paraffin-embedded lungs then were sectioned at 5-mm thickness and stained with hematoxylin and eosin. From each section, 10 random areas from 3 mice within the same group were examined by an observer unaware of the protocol at a magnification of 400×. The histopathological severity of lung injury was scored based on (1) the infiltration or aggregation of neutrophils in the airspace or vessel wall and (2) the thickness of the alveolar wall. Each assessment was graded 0, 1, 2, or 3, corresponding to no, mild, moderate, or severe injury, respectively.

### Immunohistochemistry

Formalin-fixed paraffin sections (4-µm thick) were deparaffinized before antigen retrieval, and endogenous peroxidases were blocked using 3% H_2_O_2_ in methanol for 15 min. Subsequently, the slides were incubated for 60 min with an anti-SOCS3 rabbit polyclonal antibody (1:200 dilution, Abcam, USA) and anti-WNK4 rabbit polyclonal antibody (1:200 dilution, Novus Biologicals, USA). After washing, the slides were incubated sequentially with mouse-tissue-specific horseradish peroxidase-polymer anti-rabbit antibodies (Nichirei Corporation) for 30 min. The horseradish peroxidase was reacted with the DAB substrate for 3–5 min, and the sections were then counterstained with hematoxylin. Images were captured on a microscope (DM 2500, Leica, Wetzlar, Germany) equipped with an EMCCD camera using the SPOT 4.7 advanced imaging software.

### Statistical Analysis

All results are expressed as the mean ± standard deviation (SD) and were compared using Student’s *t*-test and one-way analyses of variance. Significance was set at *P* < 0.05. The data were analyzed using SPSS software (v. 20, IBM SPSS, Armonk, NY, USA) and GraphPad Prism 5 (GraphPad Software, San Diego, CA, USA).

## Results

### VILI Is Attenuated by Obesity

**Figure 1:**
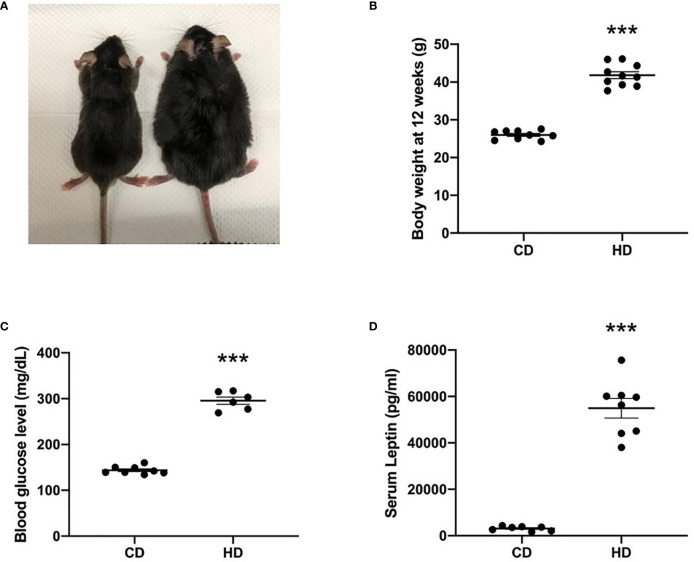
Comparison of nonobese (CD) and obese (HD) mice. **(A)** Gross appearance, **(B)** body weight at the age of 12 weeks, **(C)** blood glucose level, and **(D)** serum leptin level. Data are expressed as the mean ± SD. ^***^ *P* < 0.001 compared with the nonobese group (CD).

The comparison of nonobese with obese mice is shown in **Figure 1**. Although mice in both groups had a similar weight at the age of 3 weeks, 12-week-old mice with a high-fat diet exhibited a significantly higher body weight than did those that were fed a control diet (**Figures 1A, B**). Obese mice also have hyperglycemia (**Figure 1C**) and hyperleptinemia (**Figure 1D**), indicating the presence of glucose intolerance and leptin resistance.

Subsequently, both groups of mice were mechanically ventilated as per the protocol for 6 h (HV). Mice with spontaneous breathing (CV) were also examined, for comparison. Obese mice exhibited a trend toward a higher PIP level at the initiation of mechanical ventilation (**Supplementary Figure 1**).

**Figure 2:**
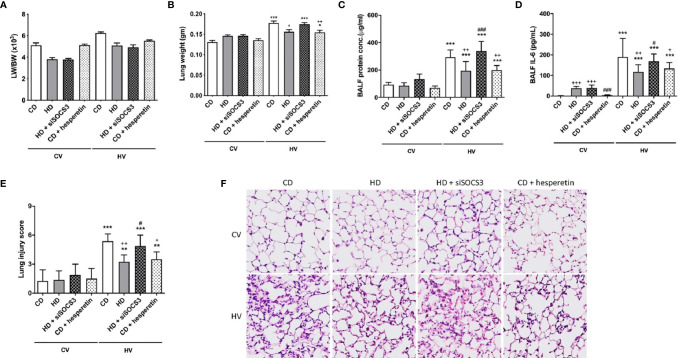
The effects of obesity and SOCS3 manipulation on ventilator-induced lung injury. These parameters were collected in nonobese mice (CD), obese mice (HD), obese mice pretreated with an intratracheal spray of a SOCS3 siRNA (HD + siSOCS3), and nonobese mice pretreated with hesperetin enterally (CD + hesperetin). They were measured after 6 h of spontaneous breathing (CV) or mechanical ventilation (HV). **(A)** Lung-to-whole-body weight ratio. **(B)** Lung weight alone. **(C)** BALF protein concentration. **(D)** BALF IL-6 level. **(E)** Lung injury score. **(F)** Hematoxylin and eosin staining of pathological specimens (magnification, 400×). BALF, bronchoalveolar lavage fluid. Data are expressed as the mean ± SD (n = 5 per group). ^*^ *P* < 0.05, ^**^ *P* < 0.01 and ^***^ *P* < 0.001 compared with the corresponding group in the CV condition. ^+^ *P* < 0.05, ^++^ *P* < 0.01, and ^+++^ *P* < 0.001 compared with the nonobese mouse group (CD). ^#^ *P* < 0.05 and ^###^ *P* < 0.001 compared with the obese mouse group (HD).

The degree of lung injury was assessed in the nonobese mice (CD) and obese mice (HD) groups (**Figure 2**) based on the gross lung-to-body weight (LW/BW) ratio, lung weight (LW) alone, the lung injury score, tissue H&E staining, and protein and interleukin 6 (IL-6) concentration in the BALF. Overall, these analyses suggested the attenuation of VILI in obese mice.

### SOCS3 Expression and the STAT3/NFκB Pathway Are Modulated in the Lung Tissues of Diet-Induced Obese Mice

**Figure 3:**
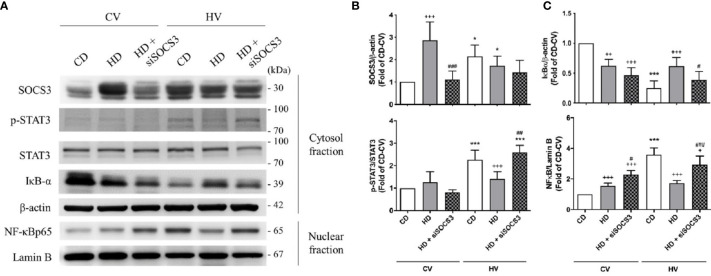
Effects of SOCS3 knockdown on the STAT3–SOCS3 and NF-κB signaling pathways. These parameters were collected in nonobese mice (CD), obese mice (HD), and obese mice pretreated with an intratracheal spray of a SOCS3 siRNA (HD + siSOCS3) after 6 h of spontaneous breathing (CV) or mechanical ventilation (HV). **(A)** Western blotting. β-Actin and Lamin B served as loading controls for cytoplasmic and nuclear proteins, respectively. Representative blots are shown. **(B)** SOCS3 and p-STAT3/STAT3. **(C)** IκBα and NF-κB. Data are expressed as the mean ± SE (n = 5 per group). ^*^ *P* < 0.05 and ^***^ *P* < 0.001 compared with the corresponding group in the CV condition. ^+^ *P* < 0.05, ^++^ *P* < 0.01, and ^+++^ *P* < 0.001 compared with the nonobese mouse group (CD). ^#^ *P* < 0.05, ^##^ *P* < 0.01, and ^###^ *P* < 0.001 compared with the obese mouse group (HD).

**Figure 4:**
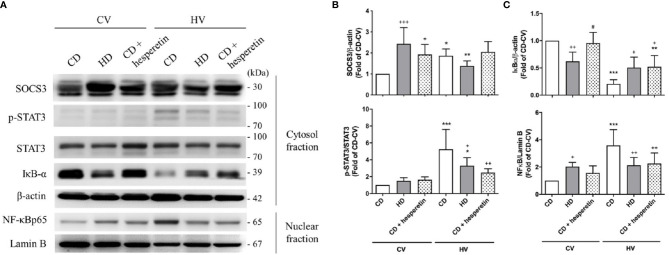
Effects of pharmaceutical SOCS3 upregulation on the STAT3–SOCS3 and NF-κB signaling pathways. These parameters were collected in nonobese mice (CD), obese mice (HD), and nonobese mice pretreated with hesperetin enterally (CD + hesperetin) after 6 h of spontaneous breathing (CV) or mechanical ventilation (HV). **(A)** Western blotting. β-Actin and Lamin B served as loading controls for cytoplasmic and nuclear proteins, respectively. Representative blots are shown. **(B)** SOCS3 and p-STAT3/STAT3. **(C)** IκBα and NF-κB. Data are expressed as the mean ± SE (n = 5 per group). ^*^ *P* < 0.05, ^**^ *P* < 0.01, and ^***^ *P* < 0.001 compared with the corresponding group in the CV condition. ^+^ *P* < 0.05, ^++^ *P* < 0.01, and ^+++^P < 0.001 compared with the nonobese mouse group (CD). ^#^ *P* < 0.05 compared with the obese mouse group (HD).

SOCS3 expression was measured in the lung tissue of mice with spontaneous breathing (CV) and was used as the baseline expression of SOCS3 before mechanical stretching in both nonobese and obese mice. The expression of the STAT3/NFκB pathway was also examined. The baseline expression SOCS3 in lung tissues was significantly higher in obese mice (**Figures 3A, B** and **4A, B**), and the STAT3/NFκB pathway was similarly overexpressed (**Figures 3** and **4**). This is consistent with well-established concept of chronic inflammation in obesity and the overexpressive SOCS3 as a negative feedback regulator.

After mechanical ventilation as a protocol (HV), the expression of SOCS3 was stimulated in nonobese mice; but in obese mice, its expression was attenuated. The STAT3/NFκB pathway appeared to be significantly activated in nonobese mice, but similarly expressed in obese mice (**Figures 3** and **4**). Moreover, if compared with nonobese mice, obese mice had marked attenuation of STAT3/NFκB signaling.

### Knockdown of SOCS3 Offset the Effect of Obesity on VILI

The expression of SOCS3 in lung tissues was knocked down by intratracheal spraying of an siRNA 48 h before the experiment. As shown in **Figures 3A, B**, obese mice exhibited significant attenuation of SOCS3 expression after siRNA knockdown, to a level similar to that detected in nonobese mice.

After mechanical ventilation, obese mice with SOCS3 knockdown had more severe lung injury compared with obese mice without siRNA treatment (**Figure 2**). The STAT3/NFκB pathway was also more activated in the former (**Figure 3**), to a level similar to that of the nonobese group. Therefore, the protective effect of obesity against VILI was significantly offset after SOCS3 attenuation.

### Pharmaceutical SOCS3 Upregulation Attenuates VILI in Nonobese Mice

For clinical relevance, we chose a pharmaceutical method to augment SOCS3 expression in lung tissues. Hesperetin, a previously proposed SOCS3 stimulator (35), was fed enterally to nonobese mice 12 h before mechanical ventilation. As shown in **Figures 4A, B**, the level of SOCS3 in lung tissues was significantly augmented in nonobese mice that were fed hesperetin.

After mechanical ventilation, the STAT3/NFκB pathway in this group was less activated, similar to that observed in obese mice (**Figure 4**). Accordingly, the severity of VILI was also attenuated (**Figure 2**). In conclusion, nonobese mice with upregulation of SOCS3 achieved by pharmaceutical stimulation had attenuated VILI, mimicking obese mice.

### Obesity Modulates the WNK4–SPAK Axis

**Figure 5:**
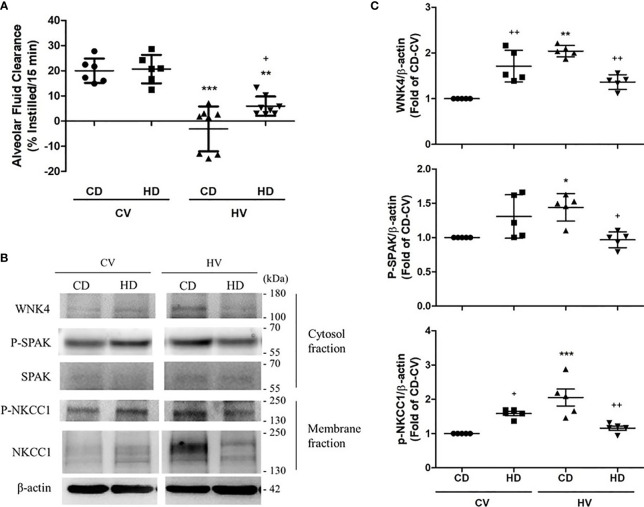
Effects of obesity on alveolar fluid clearance (AFC) and the WNK4 axis. The data were collected in nonobese mice (CD) and obese mice (HD) after 6 h of spontaneous breathing (CV) or mechanical ventilation (HV). **(A)** AFC. **(B)** Western blotting. β-Actin served as the loading control for cytoplasmic proteins. Representative blots are shown. **(C)** Expression of proteins in the WNK4 axis, including WNK4, p-SPAK, and p-NKCC1. Data are expressed as the mean ± SE (n = 5~10 per group). ^*^ *P* < 0.05, ^**^ *P* < 0.01, and ^***^ *P* < 0.001 compared with the corresponding group in the CV condition. ^+^ *P* < 0.05 and ^++^ *P* < 0.01 compared with the nonobese mouse group (CD).

Recent research has disclosed the involvement of the WNK4–SPAK pathway in lung injury. The activation of the WNK4–SPAK cascade results in the phosphorylation of epithelial NKCC1 and subsequent cotransportation of sodium and chloride ions into the alveolar space. The net response is the suppression of AFC and aggravation of lung injury. To determine whether obesity also affected WNK4 axis and AFC, we briefly examined the role of obesity in the WNK4–SPAK pathway. The AFC in the CD and HD groups is shown in **Figure 5A**. Relative preservation of AFC was observed in obese mice after the mechanical insult. Compared with nonobese mice, obese mice exhibited upregulation of WNK4, SPAK, and NKCC1 (**Figures 5B, C**) at the baseline, similar to the trend of upregulation of the STAT3/NFκB pathway observed in obese mice. After mechanical stretching, the WNK4 axis was significantly activated in nonobese mice. In contrast, the change in obese mice seemed insignificant. If compared with nonobese mice, WNK4 axis was less activated in obese mice.

### SOCS3 Slightly Upregulates WNK4 Expression but Has Lesser Role on WNK4 Axis After VILI

**Figure 6:**
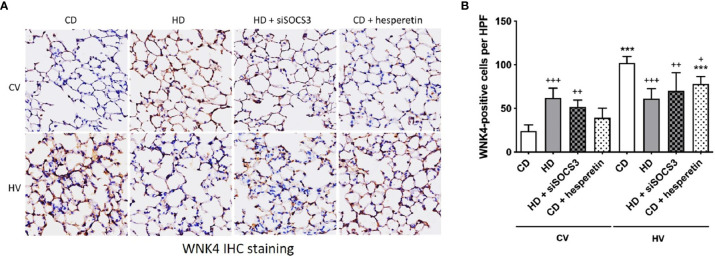
WNK4 expression in obese mice and after SOCS3 manipulation. The specimens were stained in nonobese mice (CD), obese mice (HD), obese mice pretreated with an intratracheal spray of SOCS3 siRNA (HD + siSOCS3), and nonobese mice pretreated with hesperetin enterally (CD + hesperetin) after 6 h of spontaneous breathing (CV) or mechanical ventilation (HV). **(A)** Immunohistochemical staining of WNK4. **(B)** Quantification of WNK4 expression. Data are expressed as the mean ± SE (n = 5 per group). ^***^ *P* < 0.001 compared with the corresponding group in the CV condition. ^+^ *P* < 0.05, ^++^ *P* < 0.01, and ^+++^ *P* < 0.05 compared with the nonobese mouse group (CD).

To confirm the modulation of the WNK4 axis in obese mice conferred by SOCS3, WNK4 staining was directly examined after SOCS3 manipulation (**Figure 6**) at the baseline. In obese mice with SOCS3 knockdown (HD+siSOCS3), WNK4 remained significantly upregulated (**Figure 6B**) compared with nonobese mice, despite the trend toward decreasing values. In nonobese mice, SOCS3 augmentation (CD + hesperetin) resulted in a trend toward WNK4 upregulation (**Figure 6B**), albeit to a much lesser extent compared with the effect of obesity itself. In summary, SOCS3 contributed limited-scale upregulation of the WNK4 axis.

After VILI, the activation of WNK4 in mice was slightly modulated by obesity and the manipulation of SOCS3 expression (**Figure 6**). Obese mice with initial SOCS3 knockdown (HD+siSOCS3) retained lower SOCS3 expression after the mechanical insult (**Figure 3B**) and exhibited an insignificant difference in WNK4 activation compared with obese mice (**Figure 6B**). Nonobese mice with initial SOCS3 overexpression (CD + hesperetin) retained a higher SOCS3 expression after VILI (**Figure 4B**), and showed significant WNK4 attenuation compared with nonobese mice (**Figure 6B**). Briefly, the trend in WNK change observed after VILI seemed reciprocal to the SOCS3 alteration. It’s contrary to above observation of SOCS3-yielded WNK4 upregulation.

### Obesity Overcomes the WNK4-Mediated Aggravation of VILI

**Figure 7:**
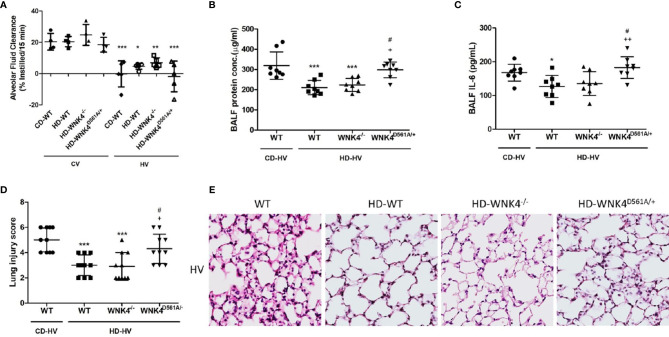
The effect of obesity and WNK4 manipulation on alveolar fluid clearance (AFC) and ventilator-induced lung injury after 6 h of mechanical ventilation (HV). These parameters were collected in nonobese wild-type mice (CD-WT), obese wild-type mice (HD-WT), obese mice with WNK4 knockout (HD-WNK4^−/−^), and obese mice with WNK4 knockin (HD-WNK4^D561A/+^). **(A)** AFC. ^*^ *P* < 0.05, ^**^ *P* < 0.01, and ^***^ *P* < 0.001 compared with the corresponding group in the CV condition. **(B)** BALF protein concentration. **(C)** BALF IL-6. **(D)** Lung injury score. **(E)** Hematoxylin and eosin staining of pathological specimens (magnification, 400×). CV, 6 h of spontaneous breathing. BALF, bronchoalveolar lavage fluid. Data are expressed as the mean ± SD (n = 10 per group, with the exception of n = 3–5 in the AFC experiment). ^*^ *P* < 0.05 and ^***^ *P* < 0.001 compared with nonobese wild-type mice (CD-WT), AFC experiment excluded. ^+^ *P* < 0.05 and ^++^ *P* < 0.01 compared with the obese wild type mice group (HD-WT). ^#^P < 0.05 compared with the obese mice with WNK4 knockout (HD-WNK4^−/−^).

To clarify the role of WNK4 in obese mice with VILI, we used mice with genetic WNK4 knockout (WNK4^−/−^) and knockin (WNK4^D561A/+^), which were generated by Yang et al. It’s also reported that WNK4^−/−^ mice exhibited low SPAK and NKCC1 phosphorylation in kidney (30, 31) and lung tissue (26, 29), whereas WNK4^D561A/+^ mice presented high SPAK and NKCC1 activity. As a result, WNK4 knockout suppressed NKCC1 phosphorylation and preserved AFC, and WNK4 knockin had the opposite effect (26, 27, 29). As shown in **Figure 7A**, obese wild-type mice had relatively preserved AFC after VILI compared with nonobese wild-type mice. Further assessment of AFC revealed that obese WNK4^−/−^ mice had values that were similar to those of the obese wild-type counterparts, whereas obese WNK4^D561A/+^ mice exhibited a significantly decreased AFC. The expression of epithelial sodium channels (ENaC) was also shown (**Supplementary Figure 5**).

**Figure 8:**
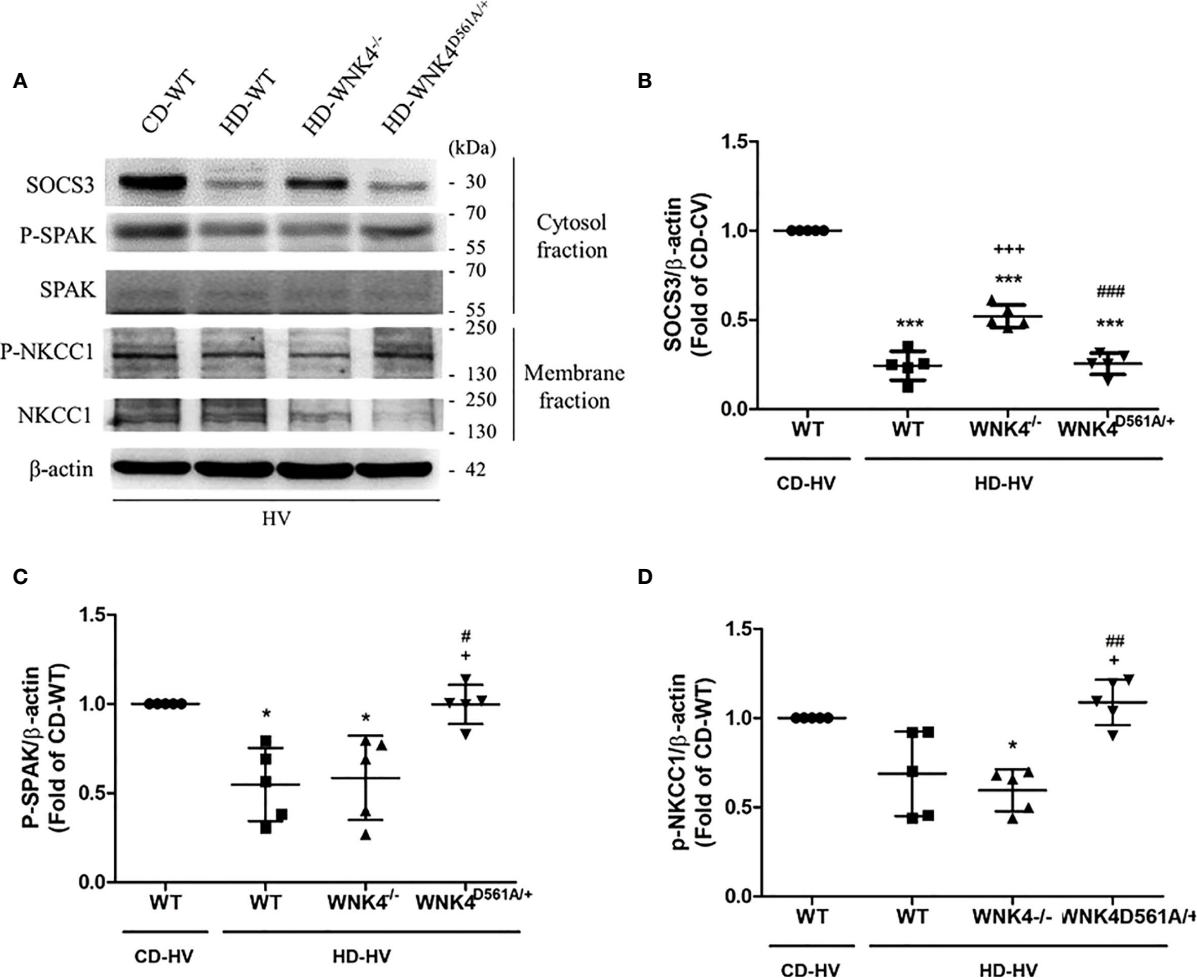
Effect of obesity and WNK4 manipulation on the SOCS3 expression and the downstream WNK4 pathway after 6 h of mechanical ventilation (HV). These data were collected in nonobese wild-type mice (CD-WT), obese wild-type mice (HD-WT), obese mice with WNK4 knockout (HD-WNK4^−/−^), and obese mice with WNK4 knockin (HD-WNK4^D561A/+^). **(A)** Western blotting. β-Actin served as a loading control for cytoplasmic proteins. Representative blots are shown. **(B)** SOCS3. **(C)** p-SPAK. **(D)** p-NKCC1. Data are expressed as the mean ± SE (n = 5 per group). ^*^ *P* < 0.05 and ^***^ *P* < 0.001 compared with nonobese wild-type mice (CD-WT). ^+^ *P* < 0.05 and ^+++^ *P* < 0.001 compared with the obese wild-type mouse group (HD-WT). ^#^ *P* < 0.05, ^##^ *P* < 0.01 and ^##^ *P* < 0.01 compared with obese mice with WNK4 knockout (HD-WNK4^−/−^).

The parameters of lung injury were also obtained, including BALF protein, BALF IL-6, lung injury score, and tissue H&E staining (**Figures 7B–E**), as well as downstream WNK4 expression (**Figures 8A, C, D**). Interestingly, SOCS3 in obese WNK4^−/−^ mice exhibited higher expression after VILI if compared to obese wild-type mice, whereas obese WNK4^D561A/+^ mice showed similar expression (**Figure 8B**). Compared with nonobese wild-type mice, lung injury was attenuated in obese wild-type obese mice, as previously stated. In turn, obese WNK4^−/−^ mice exhibited a similar VILI level to that of the obese wild-type animals. In obese WNK4^D561A/+^ mice, the level of VILI was not significantly different from that detected in nonobese wild-type mice, which was in contrast with the extreme lung injury reported previously for nonobese WNK4^D561A/+^ mice (26). Most importantly, obesity itself had a protective function against VILI and predominated over the effect of WNK4 manipulation. These results imply that the pivotal point of VILI pathogenesis is at an earlier stage of signal transduction.

## Discussion

The association between obesity and ALI has been discussed extensively, despite inconsistent conclusions. In several studies and meta-analyses, obesity was associated with higher morbidity, but with a similar or even lower mortality from ARDS (13–16). Several pathophysiological mechanisms and confounding factors related to management have been proposed to explain these clinical observations, including altered mechanics of the chest cage in patients with obesity (13), early vigilance and more attention to possible complications in these patients, a shift of macrophage polarization in adipose tissue (19), and, more interestingly, the preconditioning concept of chronic low-grade inflammation in obesity (37–40). Our study provided a novel hypothesis to explain this obesity paradox using an animal model in the context of diet induced obesity. Moreover, it is the first publication to research the role of SOCS3 in such circumstances. Noteworthy, obese mice exhibited a trend toward a higher PIP level during earlier period of mechanical ventilation. Theoretically, mechanically ventilated mice with higher PIP level were more vulnerable to barotrauma but paradoxically, obese mice with mechanical ventilation exhibited lesser severity of lung injury in our study. This finding further supports our hypothesis and strengthened the importance of signal transduction in this instance.

The suppressor of cytokine signaling 3 (SOCS3), which is a protein encoded by the *SOCS3* gene, is the downstream after initiation of the JAK2–STAT3 signaling (22). SOCS3 is upregulated in patients with obesity and impedes the signal transduction of STAT3 *via* simultaneous binding to JAK and the gp130 cytokine receptor. This negative-feedback loop accounts for leptin and insulin resistance. Concomitantly, it also hinders the initial signal transduction induced by the mechanical stress caused by ventilators. This loop of SOCS3 overexpression in obesity and resultant inhibition of STAT3/NFκB activation probably explain the attenuation of VILI observed within the first hours in our diet-induced obese mouse model. Therefore, experimental manipulation of SOCS3 expression before the mechanical insult led to a corresponding outcome. In our study, pharmaceutical stimulation of SOCS3 expression using hesperetin in nonobese mice attenuated JAK2–STAT3/NFκB signaling and subsequent lung injury. In contrast, SOCS3 siRNA-mediated knockdown in obese mice had aggravated lung injury.

Interestingly, the expression of SOCS3 after 6 h of mechanical ventilation in obese mice was paradoxically reduced, whereas a similar level of SOCS3 was observed after VILI in the siRNA- and hesperetin-treated groups. Theoretically, downstream genes of STAT3 are activated and encoded soon after mechanical stretching. This implies the immediate production of proinflammatory signals and corresponding expression of SOCS3. Under such conditions, SOCS3 is supposed to be the product of inflammation, rather than the initiator of anti-inflammation. Therefore, the level of initial SOCS3 is of greater value for lung protection than the postinsult level. Hence, it is reasonable to conclude that post-VILI SOCS3 expression in obese mice was inhibited as the result of the downregulation of the JAK2–STAT3 pathway. In the siRNA- and hesperetin-treated groups, SOCS3 expression was modulated by a STAT3-independent pathway and was not affected significantly.

The VILI was undoubtedly triggered by positive-pressure-related volutrauma and barotrauma (6, 41). Cells stretch and deform under mechanical stress, but recover and repair subsequently, after release from the stress. If the stretch exceeds the cytoprotective limit, further inflation directly leads to cell detachment from the basement membrane, epithelial and endothelial cell junction leakage, intracapillary blebs, interstitial and alveolar edema, and, finally, clinical lung injury. As a result, the VILI-associated inflammatory process is inevitably tremendous and involves almost all cell types of the lung tissue. Therefore, signal transduction at stress initiation is pivotal. Even a slight retardation of STAT3 signaling resulted in significantly different outcomes. Interestingly, previous studies concluded that preconditioning of physiological cyclic stretch-attenuated HGMB1 expression in a cell model (42) and of VILI in a rat model *via* the inhibition of the STAT3 pathway is associated with the upregulation of SOCS3 (17). Those findings also supported our hypothesis and the concept of obese preconditioning.

Hesperetin is the 4′-methoxy derivative of eriodictyol and belongs to the flavanone class of flavonoids. It is particularly enriched in lemons and oranges, and acts as an antioxidant. In cell models, hesperetin induces SOCS3 gene expression *via* a STAT3-independent process (activation of the extracellular-signal-regulated kinase [ERK]-dependent transcription factor SP3) (35). Cell-permeable forms of recombinant SOCS3 have been used to effectively suppress pathogen-induced acute inflammation (43). However, for clinical relevance and practical purposes, we chose this compound to directly stimulate SOCS3 expression. By preliminary *in vitro* (LA-4 cells) and *in vivo* (**Supplementary Figure 4**) study, the highest SOCS3 expression was detected around 12 h after hesperetin treatment. Based on this result, we concluded that hesperetin plays a role in ALI and could potentially be exploited to develop novel anti-inflammatory therapies for VILI. Moreover, similar findings were observed in other publications with either hesperetin pretreatment (44) or postinsult administration (45, 46). Hence, the role of hesperetin treatment in lung injury is significant, even though optimal timing and dosage warrants further study. Also, additive effect of hesperetin pre-treatment in obese mice with even higher SOCS3 expression could be predicted based on its STAT3-independent SOCS3 stimulation as described above. However, it still needs further experiment to confirm if corresponding protection against VILI presented.

The activation of the WNK4–SPAK pathway results in the phosphorylation of epithelial NKCC1 and subsequent cotransportation of sodium and chloride ions into the alveolar space. Moreover, it was recently established as the pivotal factor in alveolar flooding and lung edema (26, 27). In our study, higher expression of the baseline WNK4 cascade was noted in obese mice. Although this effect was limited in both groups, the hesperetin-stimulated SOCS3 increase upregulated WNK4 expression, whereas siRNA knockdown had the opposite effect. This also suggests that SOCS3 only partly accounts for the upregulation of the WNK4–SPAK cascade in obesity. The upregulation of the WNK4–SPAK pathway in obese mice at the baseline probably involved upstream factors other than SOCS3. Furthermore, reciprocal change of SOCS3 and WNK4 after VILI also implies that the VILI-associated WNK4 response likely resulted from a more dominant nonSOCS3 upstream component. Previous research has disclosed the activation of the IκB kinase–NFκB cascade-induced osmotic stress and cell swelling, which can subsequently activate WNK kinases (47, 48). Moreover, protein kinase C (PKC), which is a downstream factor of JAK2 and PI3K, has also been proposed to activate the WNK4 axis (49). In brief, upregulation of the WNK4 cascade in obesity is consistent with the condition of chronic inflammation. It probably resulted from multiple upstream pathways, including JAK2–NFκB signaling, JAK2–PI3K–PKC pathway, and, partly, SOCS3 stimulation. Notably, the expression of the WNK4 axis in obese mice after VILI was correlated with STAT3/NFκB expression, regardless of SOCS3 status. This suggests the predominance of the upstream NFκB pathway and a relatively minor role for SOCS3 in WNK4 activation.

The WNK4–SPAK–NKCC1 cascade leads to the cotransportation of sodium and chloride ions into the alveolar space. Knockout of the *WNK4* gene theoretically afforded a protective effect on pulmonary edema and eventual lung injury. However, our results showed no additional protection against VILI compared with wild-type obese mice. Our study revealed that the WNK4 axis has been attenuated in obese mice with VILI. Therefore, it is reasonable to conclude that further inhibition of WNK4 activity does not offer an additional benefit. Conversely, previous publications revealed that WNK4 knockin mice, compared with wild-type animals, sustained catastrophic lung injury in hyperoxia (26), ischemia–reperfusion (27), and lipopolysaccharide models (29). Our experiments disclosed that the severity of VILI in WNK4 knockin obese mice is only comparable to that of wild-type nonobese mice. It’s supposed the predicted aggravation of lung injury in WNK4 knockin wild-type mice is offset by the protection of obesity. In brief, the WNK4 axis represented relatively a small part of the signal transduction in VILI. Obesity provides major attenuation of NFκB signaling *via* SOCS3-associated JAK2 inhibition, and then suppresses WNK4 activity. Eventually, it overcomes the WNK4-mediated aggravation of VILI.

It’s noteworthy about the change of SOCS3 after VILI in WNK4-manipulated mice. According to our hypothesis, the decrement of SOCS3 expression after VILI reflects the attenuation of JAK2–STAT3 signaling in obese mice. In obese WNK4^−/−^ mice, the SOCS3 expression is less attenuated after VILI if compared with obese wild-type and WNK4^D561A/+^ mice. In other words, WNK4 knockout mediated attenuation of VILI leads to less severity of lung injury and inconsistent SOCS3 activity. It arises our speculation about the possibility of negative feedback loop between WNK4 axis and its upstream SOCS3. It also needs further study to determine which components of WNK4 axis are involved.

SOCS3 played an important role in obesity attenuated VILI in our study. However, the pathogenesis of lung injury comprised multiple cytokines, cells and crosstalk networks. Other than SOCS3, researchers had proposed several candidate mechanisms to explain VILI process, from molecular (42, 50, 51) to cellular level (52). For example, alveolar macrophages play a critical role in the pathogenesis of acute lung injury, whereas the polarization of macrophages are modulated by SOCS3 (19, 52). Of particular interest is the anti-inflammatory role of lipoxin A4 (LXA4). LXA4 has been widely published for its effect on attenuation of lung injury (53–56), considered *via* modulating signal transduction and alveolar fluid clearance. It also shared partial common pathway with SOCS3; and the interaction between LXA4 and SOCS3 has ever been proposed (57). In brief, SOCS3 has significant, but not unique effect on lung injury. The interaction between SOCS3 and proposed mechanism above, especially LXA4, still needs further clarification.

**Figure 9:**
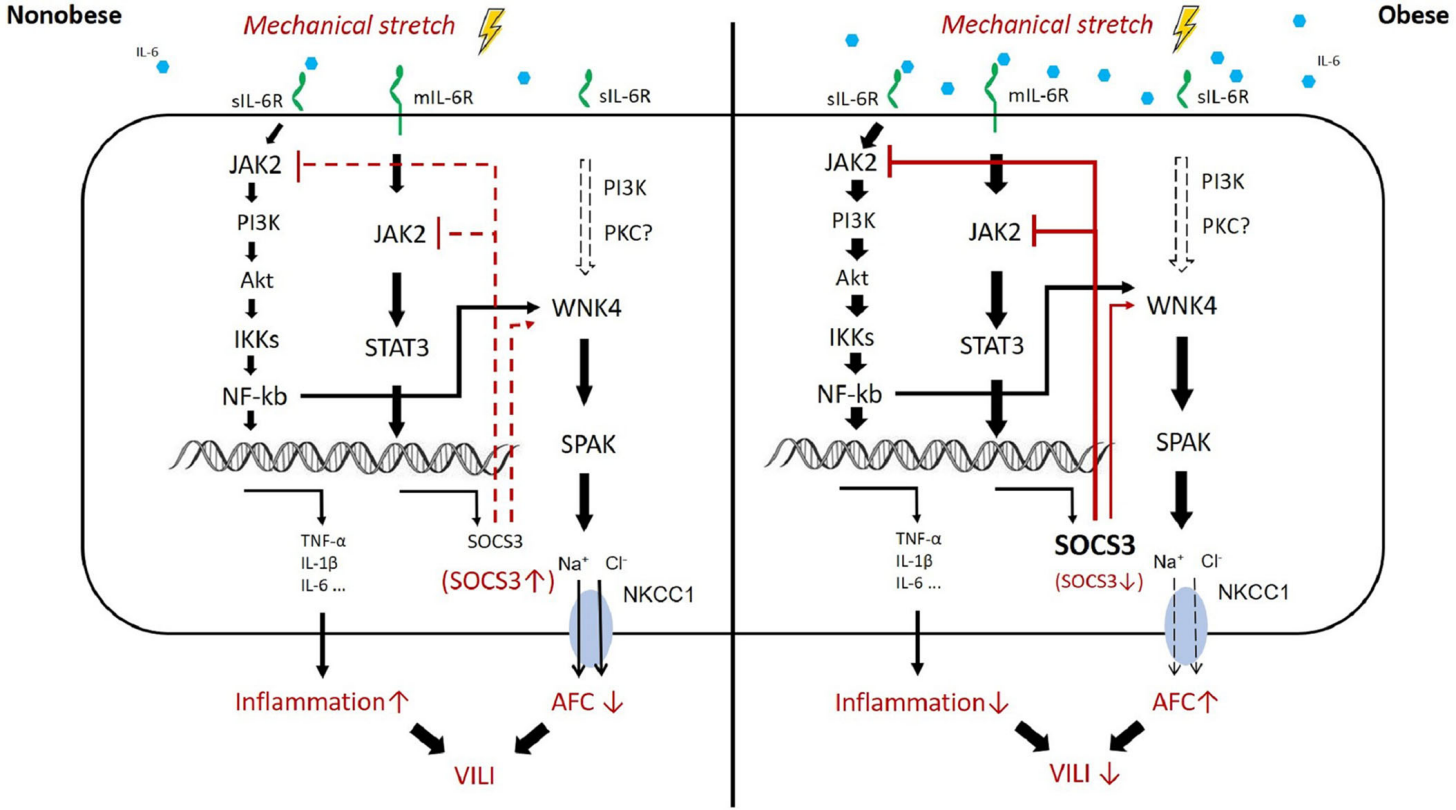
Proposed mechanism of obesity-mediated attenuation of ventilator-induced lung injury. In obesity, higher circulating levels of IL-6 result in the activation of JAK2–STAT3/NFκB and overexpression of SOCS3 at the baseline status, the so-called chronic inflammation. The upstream pathway of WNK4 includes NFκB, SOCS3, and possibly PKC. These proteins also account for the baseline upregulation of WNK4 in obesity. After mechanical stretching (brown color), signal transduction *via* JAK2 is hindered immediately by the existing SOCS3 overexpression in obesity. Subsequently, the inhibition of JAK2 signaling retards the STAT3–SOCS3 and NFκB cascades and the WNK4 axis. Eventually, VILI is also attenuated.

In summary, obesity modulated both the STAT3/NFκB pathway and the WNK4 axis (**Figure 9** and **Table 1**). SOCS3 was upregulated in obesity; it hindered the JAK2–STAT3/NFκB cascade immediately after mechanical stretching. In addition, the WNK4 axis was also modulated by several upstream, partly conferred by SOCS3. In obese mice, the WNK4 axis was significantly attenuated after VILI by the downregulation of the predominant NFκB pathway. The manipulation of WNK4 also gave the evidence of the extent in the pathogenesis of VILI. However, the interaction between SOCS3 and WNK4 axis in regulating VILI in the context of obesity warrants further investigation.

**Table 1:** Summary table of associated change between SOCS3, inflammation, removal of alveolar fluid, and VILI.

	Nonobese		Obese			
	W/T		W/T		WNK4 K/O	WNK4 K/I
	CV	HV	CV	HV	HV	HV
SOCS3	**—**	**↑↑↑**	**↑↑↑**	**↑**	**↑↑**	**↑**
BALF IL-6	**—**	**↑↑↑**	**↑**	**↑↑**	**↑↑**	**↑↑↑**
WNK4	**—**	**↑↑**	**↑↑**	**↑↑**	**—**	**↑↑↑**
AFC	**—**	**↓↓↓**	**—**	**↓↓**	**↓↓**	**↓↓↓**
VILI	**—**	**↑↑↑**	**—**	**↑**	**↑**	**↑↑↑**

## Nomenclature

STAT3/NFκB: it means simultaneous STAT3 and NFκB signaling following JAK2 activation.

## Data Availability Statement

The datasets presented in this study can be found in online repositories. The names of the repository/repositories and accession number(s) can be found in the article/**Supplementary Material**.

## Ethics Statement

The animal study was reviewed and approved by National Science Council and the Institutional Animal Care and Use Committee at the National Defense Medical Center.

## Author Contributions

S-WW, S-YW, S-ET and K-LH conceived and designed the experiments. S-WW, YW, and S-YW performed the experiments. S-WW, S-YW, S-ET and K-LH analyzed the data. C-KP and S-SY contributed reagents, materials, analytic tools. S-WW wrote the paper. All authors contributed to the article and approved the submitted version.

## Funding

This study was supported by grants from Ministry of Science and Technology (Award numbers: MOST 109-2314-B-016-029-MY3), Tri-Service General Hospital (Award numbers: TSGH-C103-087, TSGH-C105-086, TSGH-C107-076, TSGH-C108-105); and Ministry of National Defense-Medical Affairs Bureau (Award numbers: MAB-106-014, MAB-107-038).

## Conflict of Interest

The authors declare that the research was conducted in the absence of any commercial or financial relationships that could be construed as a potential conflict of interest.

## Publisher’s Note

All claims expressed in this article are solely those of the authors and do not necessarily represent those of their affiliated organizations, or those of the publisher, the editors and the reviewers. Any product that may be evaluated in this article, or claim that may be made by its manufacturer, is not guaranteed or endorsed by the publisher.

## Supplementary Material

Supplementary Figure 1 Peak inspiratory pressure (PIP) in mechanically ventilated nonobese (CD) and obese (HD) mice. It was calculated by averaging all the PIP values recorded at hour 0 (0–10 min), 3 (175–185 min), and 6 (350–360 min) after mechanical ventilation. Data are expressed as the mean ± SE (n = 10 per group). Each mice offer PIP data at hour 0, 3, 6 during 6 h of mechanical ventilation.

Supplementary Figure 2 The validation of SOCS3 siRNA efficacy in LA4 cells. Direct spray of SOCS3 siRNA was performed 48 h before the experiment started. By purchasing aliquots of all 4 individual siRNAs targeting a single gene, we choose SOCS3 siRNA-2 into *in vivo* study according to this result. β-Actin served as a loading control for cytoplasmic proteins. **P* < 0.05 compared with scramble siRNA.

Supplementary Figure 3 The validation of SOCS3 siRNA efficacy by *in vivo* evidence. This intratracheal delivery was performed 48 h before the experiment started. The RNAscope^®^ Probe- Mm-Socs3 was used for detection of target RNA within lung tissue. Hybridization signals were detected by DAB staining, followed by counterstaining with hematoxylin. **(A)** Immunohistochemistry staining of SOCS3 (brown) in lung tissue. **(B)** In situ hybridization of SOCS3 mRNA (brown dots with black arrows) in lung tissue.

Supplementary Figure 4 The choice of hesperetin dose in nonobese mice. Based on reference, the dose of 20, 40, 80mg/kg respectively were selected for comparison. Nonobese mice were fed 12 h before sacrifice. According to the result, the dose of 40mg/kg hesperetin was chosen for experiments. β-Actin served as a loading control for cytoplasmic proteins. **P* < 0.05 compared with control mice (0 mg/kg).

Supplementary Figure 5 The effect of obesity and WNK4 manipulation on the expression of ENaC subunits after 6 h of spontaneous breathing (CV) or mechanical ventilation (HV). These parameters were collected in nonobese wild-type mice (CD-WT), obese wild-type mice (HD-WT), obese mice with WNK4 knockout (HD-WNK4^−/−^), and obese mice with WNK4 knockin (HD-WNK4^D561A/+^). Data are expressed as the mean ± SE (n = 3 per group). The experiments and WB of specimens were performed separately. So, the value before and after mechanical ventilation was not comparable and for reference only. It could not reflect true relationship of ENaC expression between CV and HV group.
