# Supplementary figures and images for: Obesity Attenuates Ventilator-Induced Lung Injury by Modulating the STAT3–SOCS3 Pathway
Source: Front Immunol. 2021 Aug 20;12:720844. doi: 10.3389/fimmu.2021.720844 (PMC8417798; doi:10.3389/fimmu.2021.720844)

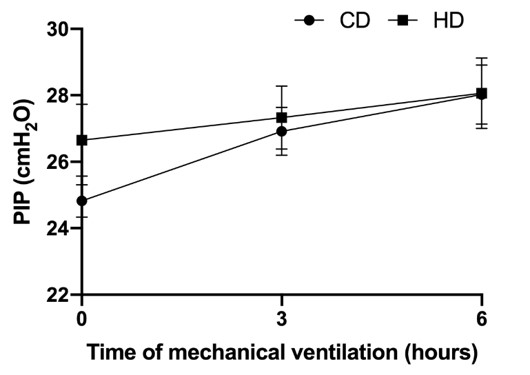

Supplement: Supplementary Figure 1 — Peak inspiratory pressure (PIP) in mechanically ventilated nonobese (CD) and obese (HD) mice. It was calculated by averaging all the PIP values recorded at hour 0 (0–10 min), 3 (175–185 min), and 6 (350–360 min) after mechanical ventilation. Data are expressed as the mean ± SE (n = 10 per group). Each mice offer PIP data at hour 0, 3, 6 during 6 h of mechanical ventilation. [file Image_1.jpeg]

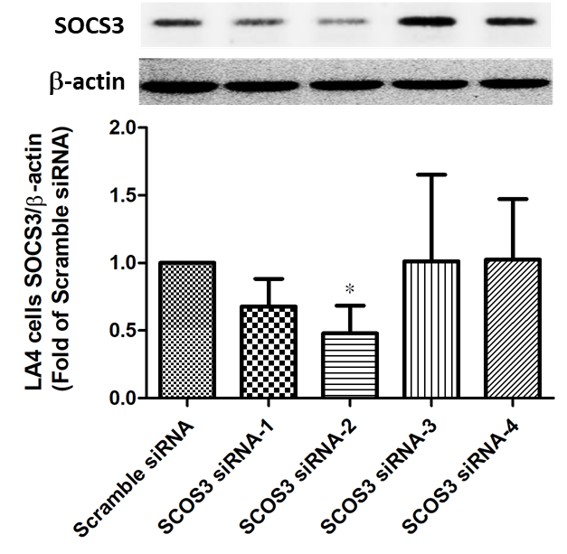

Supplement: Supplementary Figure 2 — The validation of SOCS3 siRNA efficacy in LA4 cells. Direct spray of SOCS3 siRNA was performed 48 h before the experiment started. By purchasing aliquots of all 4 individual siRNAs targeting a single gene, we choose SOCS3 siRNA-2 into in vivo study according to this result. β-Actin served as a loading control for cytoplasmic proteins. *P < 0.05 compared with scramble siRNA. [file Image_2.jpeg]

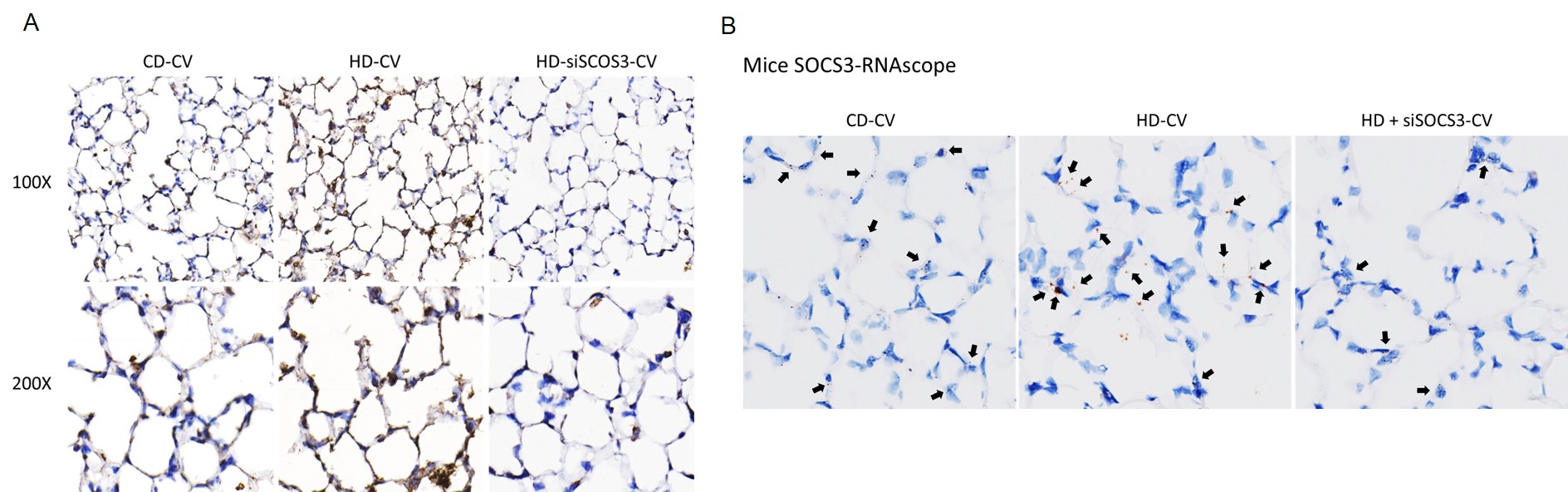

Supplement: Supplementary Figure 3 — The validation of SOCS3 siRNA efficacy by in vivo evidence. This intratracheal delivery was performed 48 h before the experiment started. The RNAscope® Probe- Mm-Socs3 was used for detection of target RNA within lung tissue. Hybridization signals were detected by DAB staining, followed by counterstaining with hematoxylin. (A) Immunohistochemistry staining of SOCS3 (brown) in lung tissue. (B) In situ hybridization of SOCS3 mRNA (brown dots with black arrows) in lung tissue. [file Image_3.jpeg]

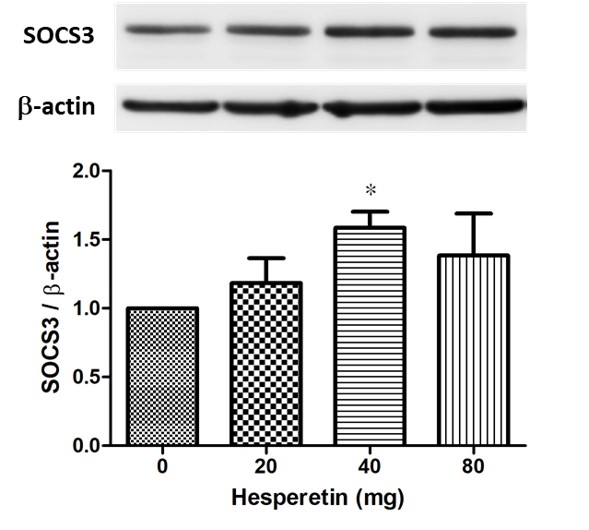

Supplement: Supplementary Figure 4 — The choice of hesperetin dose in nonobese mice. Based on reference, the dose of 20, 40, 80mg/kg respectively were selected for comparison. Nonobese mice were fed 12 h before sacrifice. According to the result, the dose of 40mg/kg hesperetin was chosen for experiments. β-Actin served as a loading control for cytoplasmic proteins. *P < 0.05 compared with control mice (0 mg/kg). [file Image_4.jpeg]

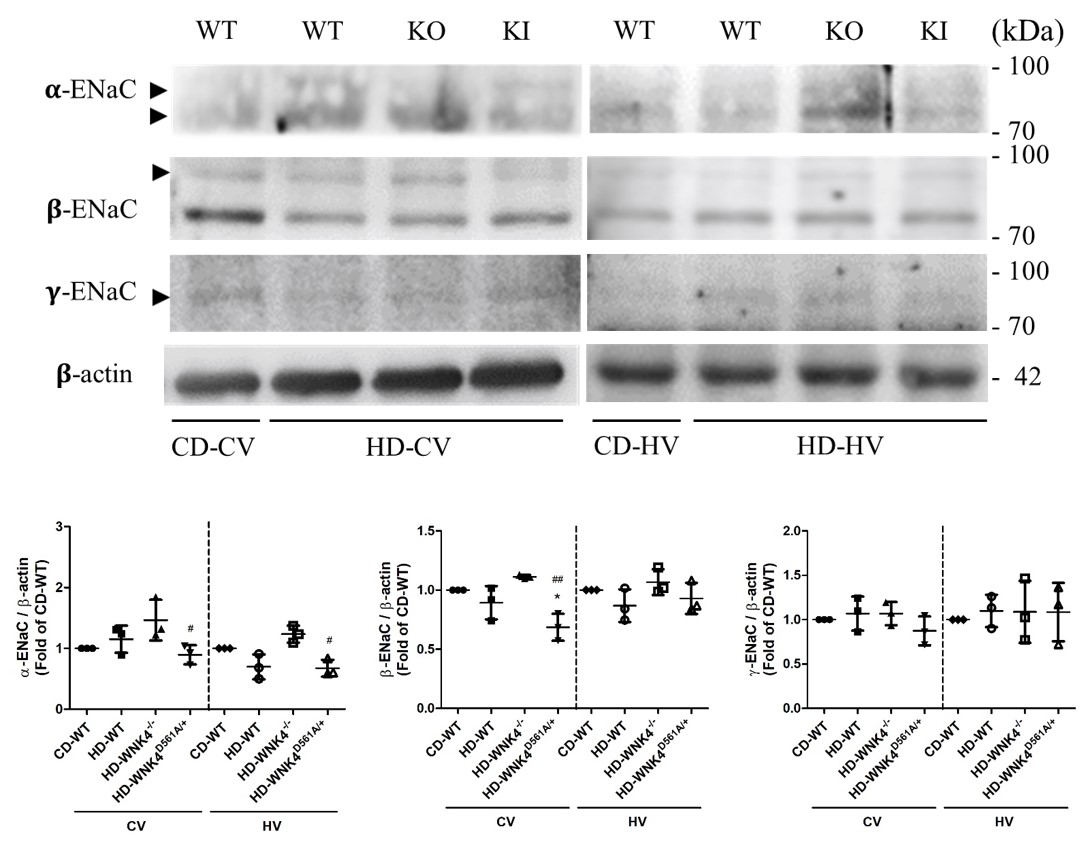

Supplement: Supplementary Figure 5 — The effect of obesity and WNK4 manipulation on the expression of ENaC subunits after 6 h of spontaneous breathing (CV) or mechanical ventilation (HV). These parameters were collected in nonobese wild-type mice (CD-WT), obese wild-type mice (HD-WT), obese mice with WNK4 knockout (HD-WNK4−/−), and obese mice with WNK4 knockin (HD-WNK4D561A/+). Data are expressed as the mean ± SE (n = 3 per group). The experiments and WB of specimens were performed separately. So, the value before and after mechanical ventilation was not comparable and for reference only. It could not reflect true relationship of ENaC expression between CV and HV group. [file Image_5.jpeg]
